# Implementation of joint health indicators in Europe - Joint Action for ECHIM. Arpo Aromaa on behalf of the ECHIM core group

**DOI:** 10.1186/0778-7367-70-22

**Published:** 2012-10-08

**Authors:** Arpo Aromaa

**Affiliations:** 1 Arpo Aromaa, National Institute for Health and Welfare, Helsinki, Finland

**Keywords:** Health indicators, Health information, European Community Health Indicators (ECHI), EU Joint Action

## Abstract

The story of the implementation of the joint EU health indicators (ECHI indicators) began in the 1990s after the Amsterdam Treaty. The first concrete step in establishing a health monitoring capacity for EU was the Commission working group set up in 1997. Several consecutive and parallel projects, notably the health indicator projects ECHI-1 and ECHI-2 between the years 2000 and 2005 led to a preparedness to implement the jointly agreed health indicators (ECHI shortlist) in all European countries. ECHIM (2005 – 2008) and the Joint Action for ECHIM (2009 - ) laid the foundation for the implementation of health indicators, and initiated Europe wide implementation proper. After the European recession of 2008 the circumstances in different countries were not optimal. Also the collaboration with the Commission could have been better. Nevertheless, the implementation process of the ECHI indicators is now well underway in most countries. By June 2012 half of the Member States had incorporated the ECHI indicators into their national health information system, and, if work can continue, by 2014 most countries are likely to have done so. Unfortunately, a gap may occur between the current programme and the next public health programme. The current momentum must not be lost. Therefore, all those responsible need to urge that the Commission (DG SANCO) together with the Member States helps to bridge the gap from June 2012 to January 2014. The new Public Health Programme provides the necessary financial instruments for setting up a permanent EU health information and reporting system.

## The past

Health indicators such as total and cause-specific mortality or infant mortality have been used in Europe for centuries. As an example, in Sweden–Finland these were supplemented by registers of births and deaths kept by parish priests. In Finland the mortality statistics were prepared by priests since 1748 recording thirty causes of death. As a matter of fact, mortality statistics served also to estimate the number of men who, with their horses, could be recruited to the King’s army. Nevertheless, actuaries in many European countries, foremost in England and Wales, were interested in mortality as a phenomenon and in particular in the distributions of diseases reflected by the mortality data. But it took a long time before these systems were modernized. In Finland, since 1936, the causes of deaths were recorded on the basis of death certificates issued by doctors.

Quite independently of each other all European countries have over the years developed their own health information systems. Of course, there was early harmonization between the Pasteur-institutes in regard of communicable diseases, starting with tuberculosis. In regard of chronic non-communicable diseases the framework provided by WHO concerning the causes of death helped to harmonize the recorded causes of death statistics. Since the late 1970s the WHO’s Health for All Programme resulted in the gathering of European health data to be archived in and disseminated from the HFA data base [1].

Also, the OECD collected its own health data set covering a large number of European and non- European OECD countries [2]. From the point of view of the European Union the most authoritative EU-collection of health statistics is that of Eurostat [3]. Nevertheless, policy relevant information on health determinants was very unevenly available. Already on superficial examination it is clear that the various international data bases use slightly different definitions and calculation methods, yielding differences between country specific figures.

The health systems and as a consequence the health information systems differ between the countries. In some systems services and medication are provided by national and regional health care providers, whereas others depend on the provision and/or coverage of costs by health insurance. However, information needs are rather similar independently of the financing system, although the availability of health data depends on the system [4].

## Emergence of EU health monitoring after the Amsterdam Treaty

The EU history proper of joint health indicators began after the Amsterdam Treaty [5], and the first concrete step was an extensive review by the Danish Ministry of Health of the health data and health indicators in Europe. Next, the Parliament asked (1997) the Commission about creating an EU health monitoring system. The Commission response was a working group set up in 1997. Its report [6] (see also the related article [7]) was presented to the Commission in 1998, and in a revised form in 2000. The report proposed setting up an EU health monitoring capacity with a network of national experts.

Instead of proceeding along the proposed lines, the Commission decided to use all its resources on time-limited project work concerning a variety of health aspects and a few non-coordinated projects on health indicators. However, also a few horizontal projects were selected for financing, and just these were responsible for developing health monitoring as a whole. In addition to funding health indicator work proper, the Commission also created an executive agency (PHEA, later EAHC) to help administer the Public Health programme.

## Public Health Programme Projects

Already before the EU Public health programmes Eurostat commissioned work on the contents and the comparability of national European health interview surveys. It was carried out by the Dutch Central Office for Statistics (Hupkens C, CBS). The researcher’s conclusion was that by year 2000 the only comparable health interview data were those on the Body Mass Index.

Firsts in the arena of horizontal Public Health programme projects were the HIS/HES projects [8] (2000 to 2003) related to gathering of health (indicator) data by surveys. They reviewed the existing national health surveys and, via recommendations, developed the methods for gathering health data by population surveys.

The true firsts of the comprehensive health indicator flagship projects were ECHI-1 and ECHI-2, carried out between 2000 and 2005 [9], [10]. The proposed ECHI shortlist covered indicators in the following fields;

A. Demographic and socioeconomic factors

B. Health status

C. Determinants of health

D. Health interventions: health services

E. Health interventions: health promotion.

The indicator work was carried out in close collaboration between various EU public health projects and MS experts. The projects reviewed the available health indicators, assessed the needs of policy relevant indicators, selected those best suited for a core set of indicators, and developed several new ones. Finally, not all recommended indicators were available in the countries. Initially, the ECHI process yielded several hundred indicators but soon there was agreement with the Commission that a more limited number of well-focused indicators should be selected for implementation. The concise set of the core indicators comprised those assessed by the majority of experts to be of high relevance for obtaining an overview of health and diseases, their occurrence, level, distribution and time trends. The ECHI –projects also initiated the development of criteria and definitions for the indicators. This was important since prior to ECHI various international data sources used different definitions and weights resulting in different indicators, which caused unnecessary confusion. The process finally led to the ECHI shortlist of 88 common health indicators, with their definitions.

A follow-up project of the survey work mentioned above and a complement also to ECHI was the HIS/HES database (later called EUHSID) [11]. Today, everybody has access to a user-friendly database comprising all national European health surveys (HIS and HES) carried out in the 2000s, and a number of national health surveys from other OECD countries.

## The ECHIM projects proper

The ECHIM projects proper were ECHIM [12] (EU health information and monitoring; 2005–2008) and the following Joint Action for ECHIM (2009 - ). The overall aim of the first ECHIM project was to lay a solid foundation for the implementation of ECHI indicators in all MSs and to initiate the implementation.

The long-term vision of ECHIM 2005 is valid also for the Joint Action:

Relevant, valid and comparable health data will be available in the EU and in most Member States

The data are transformed into valid indicators and information, which has been interpreted to meet the needs of health policy and public health

In more detail the aims and achievements of the ECHIM project (2005–2008) were the following: Further development of health indicators; Work on the definitions of indicators; Review of the availability and comparability of health indicators in international data sources (Eurostat, OECD, WHO); Assessment of the availability of indicators and health data sources; and finally, paving the way for a permanent EU Health Information System.

The main outcome of ECHIM was a thorough description of the state of affairs concerning national health information systems, health data sources and the availability of the ECHI – shortlist indicators in each of the 31 European countries.

Recently, the findings were analysed in depth [13] to assess the availability of the ECHI indicators in all European countries. The main findings were that whereas some indicators such as mortality and causes of death were universally available many other indicators were not. Many or most countries did not have national data on health determinants (including risk factors), chronic diseases and functional limitations. Therefore, many countries did not have any basis for evidence based health policy.

Examples of two topics with limited data availability were

Quality of health care and

Health promotion.

In regard of health care quality indicators 75% of the countries had data for cancer survival rates, 69% for surgical wound infections, and only 38% for diabetes control. Only half of the countries reported that they had data on equity of access.

Information on the health promotion indicators comprising e.g. policies on environmental tobacco smoke and those on healthy life styles was even poorer. Data for several of the ECHI health promotion indicators were not available. Many new developments are needed to enhance policy relevance, availability and comparability of the data sources and the indicators in the above areas.

This assessment showed that in addition to flaws at large the gathering of data and the provision of indicators in two very important areas had been almost completely neglected.

## Joint Action for ECHIM – the first three years

### A joint action in practice

In Commission theory a Joint Action is an action by the Member States, which also finance 50% of its costs. However, the Joint Action for ECHIM turned out to be a JA of the core MS institutes: they were THL (Helsinki, Finland), RIVM (Bilthoven, The Netherlands), RKI (Berlin, Germany), ISS (Rome, Italy), HI (Vilnius, Lithuania). These institutes housed the secretariats and paid 50% of the costs. The structure was:

a) ECHIM Core Group of 28 public health experts

b) ECHIM collaboration with DG SANCO expert groups, WHO Euro and OECD

c) A network comprising 1–3 health information experts in all EU and EFTA countries

The work was divided into work packages, carried out by each of the secretariats. In brief, they were the following: ECHI Indicators; Website for the indicators; Implementation of the indicators (Northern and Western MSs and Eastern and Southern MSs), Data flow.

The expected results comprise a new release of the ECHI shortlist, the ECHIM products website [14], MS and EU specific guidelines for indicator implementation, improved data flow, the electronic presentation of the health data based on the ECHI shortlist in HEIDI [15], the first joint analyses on data, and the final report.

Overall progress has been rather good, and by June 2012 the majority of these goals have been reached. One can also judge that in comparison with the original expectation of a duration of 6 years for the complete implementation process, progress has been faster than expected. Nevertheless a few more years are needed to create a full-fledged information system.

## Survey data needed

To allow for the effect of the expected different national situations and developments the original ECHIM plan was based on reserving sufficient time and reasonable additional resources for improving the present health information systems. Most countries needed to improve the gathering of survey data. The foremost task was to develop the European Health interview Survey (EHIS) in collaboration with all countries. ECHIM and EHIS worked very well together in ensuring that new core health indicators based on interview survey data, become available.

Next, a number of data are needed, which can only be obtained by comparable national health examination surveys (EHES). Without health examinations, important policy relevant information remains lacking. Examples are data on topics such as high blood pressure, high serum lipids, diabetes control, other biochemically determined blood constituents, body mass index, functional limitations and the treatment situation.

Without European progress in these surveys, there is no chance to add to the national health information systems the indicators now lacking. Therefore, both the European health interview survey (EHIS wave 2) must be further developed and an entirely new national health examination survey system (over 70% of the countries had none) must be set up. Taken together more than seven years have now been needed to develop both the surveys and the health indicator system. This can be compared with the view of an expert assessing the ECHIM 2005 proposal who suggested that one year should suffice for implementing the joint health indicators! It is often stated, that register data would be the best choice for valid and comparable European health indicators. However, it is well known that health data drawn from hospital discharge registers are comparable only between some 10 countries. Large comparability problems are also known to exist between the sickness insurance and use of medicines registers as well as primary health care registers. ECHIM is an example of social engineering in 31 countries. Much further work is needed to improve the availability of the data.

In order to make things move it would have been good to be able to provide at least some symbolic support for the country experts expected to do the job. Neither the experts evaluating the ECHIM plan nor DG SANCO and PHEA/EAHC appreciated that there was a great need to encourage the national input into the implementation. Thus, none of the financing proposed to support national experts in the countries, was allocated. Some financial support for the countries would have greatly speeded up the implementation process.

## Progress of the Joint Action for ECHIM

The Joint Action for ECHIM was an immediate follow-up of the ECHIM (2005–2008) project and one could have expected that previous work would have been smoothly continued in this next phase. Nevertheless, some time was needed to get implementation work going in the countries. After joint planning by the secretariats started in the spring 2009, work in the MSs started with a delay of a few months. The five co - ordinating secretariats (THL, Helsinki; RIVM, Bilthoven; RKI, Berlin; ISS, Rome; HI, Vilnius) prepared guidelines for the implementation of ECHI indicators. According to them a national implementation team should be set up in each country. During the first year good progress was made in the formal organization of the work in most countries. Nevertheless, it became soon evident that there would be considerable variation of the progress between the countries. The differing points of departure, the different impact of the recession of 2008, and the different national priorities played a role.

The other big tasks were to complete the list of health indicators and their definitions and to improve the flow of data and their dissemination. Unfortunately, it became evident, that there was a silent controversy between DG SANCO and JA for ECHIM on the IT-solution about gathering the data in a central repository and in disseminating them. The background for this was that ECHIM had intended to use the Dutch EUPHIX- system for this work whilst DG SANCO decided to create a proprietary system.

## The near future

In 2011 ECHIM presented to the Commission a document about a sustainable future for ECHI [16]. The paper was based on the expectation that the Commission would be positively inclined toward supporting the ECHIM process.

If ECHIM work continues, we can expect that a complete joint European health information and indicator system is in place in most countries by 2014. Then the next step under the new Public Health Programme would be to set up a full-fledged European health indicator and reporting system. The tasks of such a system were initially outlined in the working group report of 1998.

Doubt was caused by discussions during a meeting between ECHIM and SANCO in December 2011. The Commission representative stated that DG SANCO will not support the ECHIM process. If that position holds, JA for ECHIM will continue until 30.6.2012, and after that no further steps are foreseen. That approach endangers the need to bridge the time from 30.6.2012 to the new programme beginning in 2014. Stopping ECHIM now would be a disaster both for the Commission, the Member States and health monitoring in Europe.

After the best MS experts have worked successfully for 15 years toward a joint European health indicator system there is a threat that work is stopped just when the goal is about to be reached. I can only urge that the Commission continues to support the present ECHIM work so that a permanent health information and reporting system can be set up in 2014.

## Collaboration with the Commission

The ECHIM leadership expected that the JA for ECHIM would be carried out in good collaboration with the Commission. As a matter of fact, in technical everyday matters the co-operation with the Commission was good. A positive example was that the Commission agreed to enlarge the core group meetings held once a year to so called extended core group meetings, enabling experts from all European countries to participate once a year in Luxembourg.

Unfortunately, some problems disturbed the collaboration in policy relevant areas. A letter of encouragement from SANCO to the Member States was delayed by two years. Second, the parallel SANCO action concerning the central data base interfered with the planned ECHIM activity on this topic.

## A future for EU health information?

Not unexpectedly, it soon became clear that the JA for ECHIM was about to lead to a permanent health monitoring system. But that holds only under the prerequisite that the current secretariats and personnel can continue to work after June 2012. As mentioned above in 2011 ECHIM prepared a document on the future and suggested that the Commission should help to bridge the gap from June 2012 to the next Public Health programme.

## Achievements by the end of June 2012

17 countries reported by the end of September 2011 that they had a national implementation plan in place, and 23 countries had participated in the Pilot Data Collection. However, only 8 countries had implemented the ECHI indicators as part of their national health information system. Those countries had a set of ECHI indicators in their national health data base. By the end of June 2012 half of the Member States had included the ECHI shortlist indicators and several more stated that they were in the process of doing so.

From the point of view of all the Member States, the Commission and European health monitoring at large, nothing is more important than to retain the present momentum.

ECHIM expects that despite of the progress, it will take several more years to implement ECHI indicators in all countries implying that the full implementation in most European countries will be achieved by 2014 – 2015. Nevertheless, a health information system is never ready. After the initial goals have been reached, work needs to concentrate on the improvement of the indicators, obtaining proof about their validity and comparability, assessing repeatedly the policy relevance, and ensuring that the whole system for gathering and disseminating the data and their interpretations is fully functional. A European central health monitoring capacity also needs to take care of health reporting, together with the national counterparts. Finally, there is a constant need to improve the ability of the national experts and other users to utilize the data and indicators to their best. Several of these tasks could best be carried out jointly by an EU center and by a number of high quality national Public Health Institutes. At EU level the collaboration in health monitoring and reporting between EU, WHO and OECD should also be enhanced. In particular, the same core indicators should be used in all European countries.

Understandably, not all countries are equally devoted to use the shortlist exactly as presented. Instead, they prefer slightly modified indicator sets. This is due to that not all indicators were equally relevant and that valid data for some of them cannot be obtained in many countries. The shortlist comprises a few indicators, which cannot be obtained by present means. On the other hand, some important indicators are not included in the list. An important example is the blood lipid levels, which should be added to the present short list. Finally, it is to be expected that other changes may occur quite quickly in the needed indicator set. All this draws attention to the fact that the present version of the ECHI list requires repeated upgrades.

## Looking back fifteen years

During the past fifteen years numerous high level health monitoring experts have put in their best knowledge and used a lot of time to improve health indicators and monitoring. Looking back to the beginning [6] in the late 1990s we have achieved a lot by voluntary collaboration. The amount and value of these resources far exceeds the financial input of the Commission. Therefore ECHIM really has been an endeavor by the countries for the countries. In this situation the views and wishes of the Member States must bear most of the European weight. The only reasonable outcome is that the Commission ensures that ECHIM work can continue and that its outcome, the permanent EU health information and reporting system, is established. The practical constructive solution is that the Commission provides further limited financial support, to enable the ECHIM network and the Member States to finalize the European Health Monitoring system.

## Competing interests

The author declares that he has no competing interests.
